# Assessment of Knowledge, Perceptions, and Attitudes During the Global Mpox Outbreak in June 2022: A Cross-Sectional Study From the United Arab Emirates

**DOI:** 10.3389/ijph.2023.1606080

**Published:** 2023-11-06

**Authors:** Rouba Karen Zeidan, Ankita Shukla, Amal Hussein, Hamzah AlZubaidi, Mohamad-Hani Temsah, Mohamed S. AlHajjaj, Najlaa Al-Bluwi, Manal Awad, Hawra Ali Hussein Alsayed, Narjes Saheb Sharif-Askari, Zahraa AlHano, Razan Agha, Qutayba Hamid, Rabih Halwani, Basema Saddik

**Affiliations:** ^1^ Research Institute for Medical and Health Sciences, University of Sharjah, Sharjah, United Arab Emirates; ^2^ Department of Family and Community Medicine and Behavioural Sciences, College of Medicine, University of Sharjah, Sharjah, United Arab Emirates; ^3^ College of Pharmacy, University of Sharjah, Sharjah, United Arab Emirates; ^4^ School of Medicine, Deakin Rural Health, Deakin University Faculty of Health, Warrnambool, VIC, Australia; ^5^ Department of Pediatrics, College of Medicine, King Saud University, Riyadh, Saudi Arabia; ^6^ Department of Clinical Sciences, College of Medicine, University of Sharjah, Sharjah, United Arab Emirates; ^7^ Department of Preventive and Restorative Dentistry, College of Dental Medicine, University of Sharjah, Sharjah, United Arab Emirates; ^8^ Department of Pharmacy, Dubai Academic Health Corporation, Dubai, United Arab Emirates; ^9^ College of Medicine, University of Sharjah, Sharjah, United Arab Emirates; ^10^ Meakins-Christie Laboratories, Research Institute, McGill University Health Center, Montréal, QC, Canada; ^11^ School of Population Health, Faculty of Medicine, University of New South Wales, Sydney, NSW, Australia

**Keywords:** mpox, outbreak, perception, attitude, stigma

## Abstract

**Objectives:** To examine knowledge, worry, anxiety, and vaccine acceptance for mpox among UAE adults.

**Methods:** An online survey, advertised on academic and social media platform in June 2022 collected data from 959 participants (aged 18 and above) on mpox beliefs, risks, knowledge, worry, anxiety, COVID-19 infection, vaccination, and willingness to receive the mpox vaccine. Bivariate and logistic regression analysis identified associations and predictors between variables.

**Results:** 56% had optimal knowledge of mpox transmission and symptoms. 54% were worried, and 27% experienced anxiety related to the outbreak. Knowledge scores were higher among women, healthcare workers, and those with reliable information sources. High perceived infection risk, changes in precautionary measures, and belief in difficult treatment predicted more worry and anxiety. Higher worry and two or more doses of the COVID-19 vaccine predicted higher likelihood of taking the mpox vaccine.

**Conclusion:** The UAE population showed low knowledge and high worry and anxiety during the global mpox outbreak. Increasing public awareness through targeted educational campaigns is vital. Promoting better understanding of infectious diseases, addressing concerns, and encouraging vaccine uptake can prepare for future outbreaks.

## Introduction

Mpox previously known as monkeypox [1] has been sporadically documented in small outbreaks in the past. However, for the first time in May 2022, the World Health Organization (WHO) received reports from 12 member states that were previously non-endemic for the virus [2]. The United Arab Emirates (UAE) was the first country in the Middle East and North Africa (MENA) region to report a mpox case on 24th May 2022. As per WHO, there were 16 confirmed mpox cases in the UAE, with the last reported case being on the 24th of July 2022 [3]. However, a recently published paper from two communicable disease centres in Abu Dhabi, UAE, reported a total of 176 confirmed mpox cases during May to December 2022 [4]. It is worth noting that there have been no formal reports published by the Ministry of Health and Prevention (MOHAP) regarding these numbers or any updated figures from other emirates.

Globally, a majority of reported mpox cases (96.3%) were among males, with a high proportion (84.1%) identifying as men who have sex with men. In most cases (82.1%), physical contact during sexual activity was presumed to be the mode of transmission, and approximately half of the cases (52%), also had HIV co-infection along with mpox [3]. The only clinical study available for the UAE found similar clinical characteristics of the disease as observed in other countries [4]. The infection was dominant among young males, with a median age of 30 years, representing 93% of the cases. Though, this study also found sexual exposure to be a significant factor in mpox infection cases but noted a reluctance among patients to disclose this information.

With coronavirus disease-19 (COVID-19) still prevalent worldwide, healthcare regulators swiftly initiated efforts to increase awareness about the mpox disease and actively began developing and disseminating precautionary protocols. However, the mpox outbreak quickly attracted news and media reports, leading to the spread of misinformation and conspiracy beliefs [5–7]. The WHO has warned that misinformation about mpox is spreading more rapidly than the mpox virus (MPXV), which poses a challenge to preventing its rapid spread [8].

As seen in the response to other infectious diseases, e.g., - during the COVID-19 pandemic, mpox has also witnessed reports of stigma and discrimination [9, 10]. The evidence suggesting sexual contact as the primary mode of transmission, particularly among gay, bisexual, and other men who have sex with men [11], is likely to further alleviate the stigma and discrimination associated with the disease as was the case while combating other sexually transmitted infections such as HIV/AIDS.

Previous outbreaks of infectious diseases, such as severe acute respiratory syndrome (SARS), influenza, and COVID-19 have been linked to various psychosocial effects [12–16]. A study conducted during the COVID-19 pandemic, in the UAE concluded that almost 71% of participants were anxious, with 38% reporting moderate to severe anxiety [14]. Consequently, the potential spread of misinformation regarding mpox could lead to similar fear and anxiety among the UAE population. However, there remains a lack of available data on the knowledge and awareness of mpox in the country. As mentioned earlier, even the reporting of cases differs between MOHAP platforms and individual research studies. This lack of comprehensive data may hinder the country’s ability to fully understand the epidemiology of mpox within its borders and subsequently in the development of effective public health programs.

To mitigate this knowledge gap, this study aims to examine the knowledge of mpox symptoms and transmission as well as anxiety levels among the adult population in the UAE. Additionally, the study seeks to identify the factors associated with awareness, worry, anxiety, and vaccine acceptance related to mpox.

## Methods

### Study Design and Sample Size

An online cross-sectional survey was conducted among adult UAE citizens/residents aged 18 years and above for 2 weeks from 7th to 23rd June 2022 using the SurveyMonkey platform. The survey was advertised on various academic and social media platforms, including LinkedIn, Facebook, WhatsApp, university portals, and email. Using the snowball technique, all adults over the age of 18 years, living in the UAE and who could read and understand Arabic or English were invited to complete the survey.

Participants who did not meet these inclusion criteria were excluded from the survey. To ensure a 95% confidence level with a 5% margin of error and an expected prevalence of 50% (due to limited data on the prevalence of mpox awareness in the UAE), a minimum sample size of 385 was calculated. Considering incomplete surveys and non-responses, the required sample size was increased by 20%, making the minimum number of participants 462.

The online survey was accessed and submitted by 1080 individuals, 19(1.8%) refused to participate outright and 102(9.4%) did not complete the survey although consenting to participate. The survey completion rate was 88.8%.

### Survey Tool

A validated and structured self-administered questionnaire comprising 36 questions was used to collect data on knowledge, worry, and anxiety levels related to the recent mpox outbreak. The questionnaire was adapted from previous studies on anxiety and stress due to COVID-19 [12, 13] and included multiple-choice, true or false questions, and 5-item Likert scales. It was prepared in English and translated into Arabic by an expert from the research team, with back-translation to ensure equivalence reliability. The survey was piloted among ten adults and sent to a group of five experts for content validation. Amendments were made based on their recommendations. The online questionnaire took about 10 minutes to complete and had several sections including:

(i) Demographics - participants provided information on their age, sex, educational level, ethnicity, marital status, level of schooling, and employment status.
(ii) Beliefs and perceived risks related to mpox - participants responded to statements on whether they believed they had sufficient information on mpox transmission route, disease progression, signs, symptoms, treatment and perceived risk using five 5-point Likert scale (strongly agree “1” to strongly disagree “5.” For analysis, the responses were later recategorized into two groups. Participants who selected “strongly agree” or “agree” had their responses coded as “1,” while those who chose any other option had their responses coded as “0.”
(iii) History of COVID-19 infection and vaccination - participants reported their previous COVID-19 infection (no, yes with no/mild symptoms and yes with moderate/severe symptoms) and COVID-19 vaccination status (less than two doses, only two doses, and booster dose).
(iv) Knowledge of mpox transmission routes and signs and symptoms - participants answered “true,” “false,” or “do not know” on 19 statements related to mpox transmission and signs and symptoms. A composite score was calculated, representing the sum of correctly answered statements, with values of 0 showing the lowest knowledge level, and 19 the highest. This score was later categorized into dichotomous categories through a standard median split (median = 12). A score of ≥12 indicated a value above the median, while scores below 12 represented values below the median. For practical purposes, we will refer to the latter group as having a low knowledge score, and individuals scoring ≥12 as having a high knowledge score throughout the manuscript.
(v) Worry due to mpox outbreak - participants rated their levels of worry due to the recent mpox outbreak using five 5-point Likert scale questions (not worried at all “1” to extremely worried “5”). A worry score was calculated by adding the responses to the five questions (minimum of 5 and maximum of 25, the highest worry level. A standard median split was carried out with a median value of 13.5. A score below 13.5 is indicative of lower worry scores, while scores of 13.5 or higher show higher worry scores compared to the median. For practical purposes, we will refer to the latter group throughout the manuscript being more worried.
(vi) Anxiety - the generalized anxiety disorder scale (GAD-7) was used. GAD-7 is a self-reported 7-item validated scale that measures anxiety [17]. The scale consists of responses ranging from 0 (not at all) to 3 (nearly every day). A composite score was calculated for each participant. A score of 8 or more has been identified as a screener for panic disorder, social phobia and PTSD (with sensitivity of 77% and specificity of 82%) [18], and this cut-off value has been previously used in the UAE [14]. In this study, participants were categorized using the above-mentioned cut-off point.

We measure both worry and anxiety here because while they may seem similar, there are distinct differences between the two. A study by Gana et al. aimed to determine whether worry and anxiety are the same or different constructs. The association between anxiety and worry is not bidirectional. Worry has a substantial effect on anxiety, but there is no significant effect in the opposite direction. Also, anxiety was directly linked to depression, while worry does not directly impact depression. Instead, the relationship between worry and depression is mediated by anxiety [19].

(vii) Change in behavior due to the monkeypox outbreak-participants were asked to select options applicable to them related to behavior change (restricted my social contacts, cautious to travel to another country, using protective measures like masks and sanitizers more seriously, and concerned about attending big gatherings or crowded places)
(viii) Likelihood to take mpox vaccine if available - participants were asked to rate their willingness to take the mpox vaccine on a 5-point Likert scale (ranging from very likely to very unlikely), both for themselves and for their family members. For analysis, the responses were regrouped as 1 for “very likely or likely” else “0”.

Participants were provided with information about the aims and purpose of the study and were asked to consent to their participation before proceeding to complete the online survey.

### Statistical Analysis

Data collected in SurveyMonkey were exported to excel and then analysed using SPSS software, version 28 (IBM Corp., Armonk, NY, USA). Descriptive analyses were demonstrated as frequencies and percentages. Figures present the percentage of responses related to sources of mpox information, perceptions of knowledge, perceived risks, changes in behavior due to the mpox outbreak, and likelihood to take the vaccine if available. The Pearson Chi square (χ2) test was used to examine the association between the selected dependent variables (knowledge of mpox disease, worry and anxiety due to the recent mpox outbreak) and the independent variables (social demographic characteristics, previous COVID-19 infection status, smallpox vaccination, and whether the participants believed- (i) they were at risk of being infected by mpox, (ii) mpox may have consequences on their health and that (iii) mpox is difficult to treat). Four binary logistic regressions were conducted to identify factors that could explain the variations in the levels of knowledge, worry, anxiety, and the likelihood to take the mpox vaccine. For each dependent variable, all independent variables that showed significant associations in the bivariate analysis were entered into the regression model. A backward elimination was used, and adjusted odds ratios (AOR) and their associated 95% confidence intervals were reported. The statistical level of significance was set at 0.05.

### Ethical Approval

The study was approved by the University of Sharjah’s Research Ethics Committee (REC-22-05-26) in June 2022.

## Results

A total of 959 complete responses were collected from the online survey. The majority of participants were aged between 18 and 24 years (40%), were females (71%), and of Arab ethnicity (71%). Most respondents were engaged in work unrelated to healthcare, with 20% being healthcare workers (HCWs). The respondents’ median knowledge score was 12, and 43.8% of the respondents had scores of 11/19 or lower, categorized as having low knowledge levels (<58% of correct answers). The median worry index was 13.5 for a total of 25. In addition, 14.7% had moderate or severe anxiety as per GAD-7 scores. When asked about how difficult the impact of the different GAD items on participants personal and work life was, 41.6% of participants reported the related problems as somewhat difficult, while 9.7% found them to be very/extremely difficult.

Participants were also inquired about their previous encounters with COVID-19 as well as their vaccination status, aiming to examine potential associations between past experiences with outbreaks, the current mpox outbreak, and participants’ willingness to get vaccinated. More than half (54%) of respondents reported they never had COVID-19, and among those who had been previously infected, 21% had no/mild symptoms and 25% reported having moderate/severe symptoms. The majority of participants reported they had taken the booster dose of the COVID-19 vaccine (63%), 18% reported having only two doses, and a mere 4% reported not taking any COVID-19 vaccine (Table 1).

**TABLE 1:** Sociodemographic and other background characteristics of the study population (N = 959). Assessment of knowledge, perceptions and attitudes during the global Mpox outbreak in June 2022: A cross-sectional study from the United Arab Emirates, United Arab Emirates, 2022.

		n	%
Age	18–24	384	40.0
	25–34	238	24.8
	35–44	188	19.6
	45+	149	15.5
Gender	Male	278	29.0
	Female	681	71.0
Marital status	Single	505	52.7
	Married	436	45.5
	Widowed/Divorced	18	1.9
Occupation	Others	767	80.0
	Healthcare Worker	192	20.0
Education	High school or less	219	22.8
	Undergraduate	487	50.8
	Postgraduate	253	26.4
UAE National	No	717	74.8
	Yes	242	25.2
Ethnicity	Non-Arab	279	29.1
	Arab	680	70.9
Ever received smallpox vaccine	No	276	28.8
	Yes	293	30.6
	Don’t know	251	26.2
	Missing	139	14.5
Behavioral change following the mpox outbreak	Restricted social contacts	101	12.3
	Adopted protective measures	266	32.4
	Concerned with gatherings	202	24.6
	Cautious about traveling	171	20.9
	No change although concerned	265	32.3
	No change because not concerned	248	30.2
Knowledge score (Mean = 11.6 ± 4.9; Median = 12)	Score <12.00	372	43.8
	Score 12.00+	478	56.2
Please rate how worried you are about catching mpox yourself?	Not worried	165	19.6
	Little/Somewhat worried	491	58.5
	Very/Extremely worried	184	21.9
If you are a healthcare provider, how worried are you about transmitting mpox from your work to one of your family members/friends?	Not worried	21	11.7
	Little/Somewhat worried	91	50.8
	Very/Extremely worried	67	37.4
How worried are you about the possibility of being isolated from your family	Not worried	172	20.5
	Little/Somewhat worried	332	39.5
	Very/Extremely worried	336	40.0
How worried are you about the possibility of a complete lockdown again	Not worried	180	21.4
	Little/Somewhat worried	298	35.5
	Very/Extremely worried	362	43.1
How worried are you about the possibility of losing your/your family members’ jobs as people did during the COVID-19 outbreak	Not worried	200	23.8
	Little/Somewhat worried	269	32.0
	Very/Extremely worried	371	44.2
Worry index (Median = 13.5)	<13.50	420	50.0
	13.50+	420	50.0
Presence of GAD (GAD ≥ 8)	No	667	80.5
	Yes	162	19.5
GAD severity	Minimal anxiety	525	63.3
	Mild anxiety	182	22.0
	Moderate anxiety	77	9.3
	Severe anxiety	45	5.4
How difficult have items in the GAD score made it for you to do your work, take care of things at home, or get along with other people?	Not difficult at all	385	48.7
	Somewhat difficult	329	41.6
	Very/Extremely difficult	77	9.7
Previous COVID-19 infection	No	519	54.1
	Yes, with no/mild symptoms	198	20.7
	Yes, with moderate/severe symptoms	242	25.2
COVID-19 vaccination history	Less than two doses	39	4.1
	Only two doses	170	17.7
	Booster dose	605	63.1
	Missing	145	15.1
Total		959	100

UAE, United Arab Emirates; GAD, generalized anxiety disorder.

**FIGURE 1:**
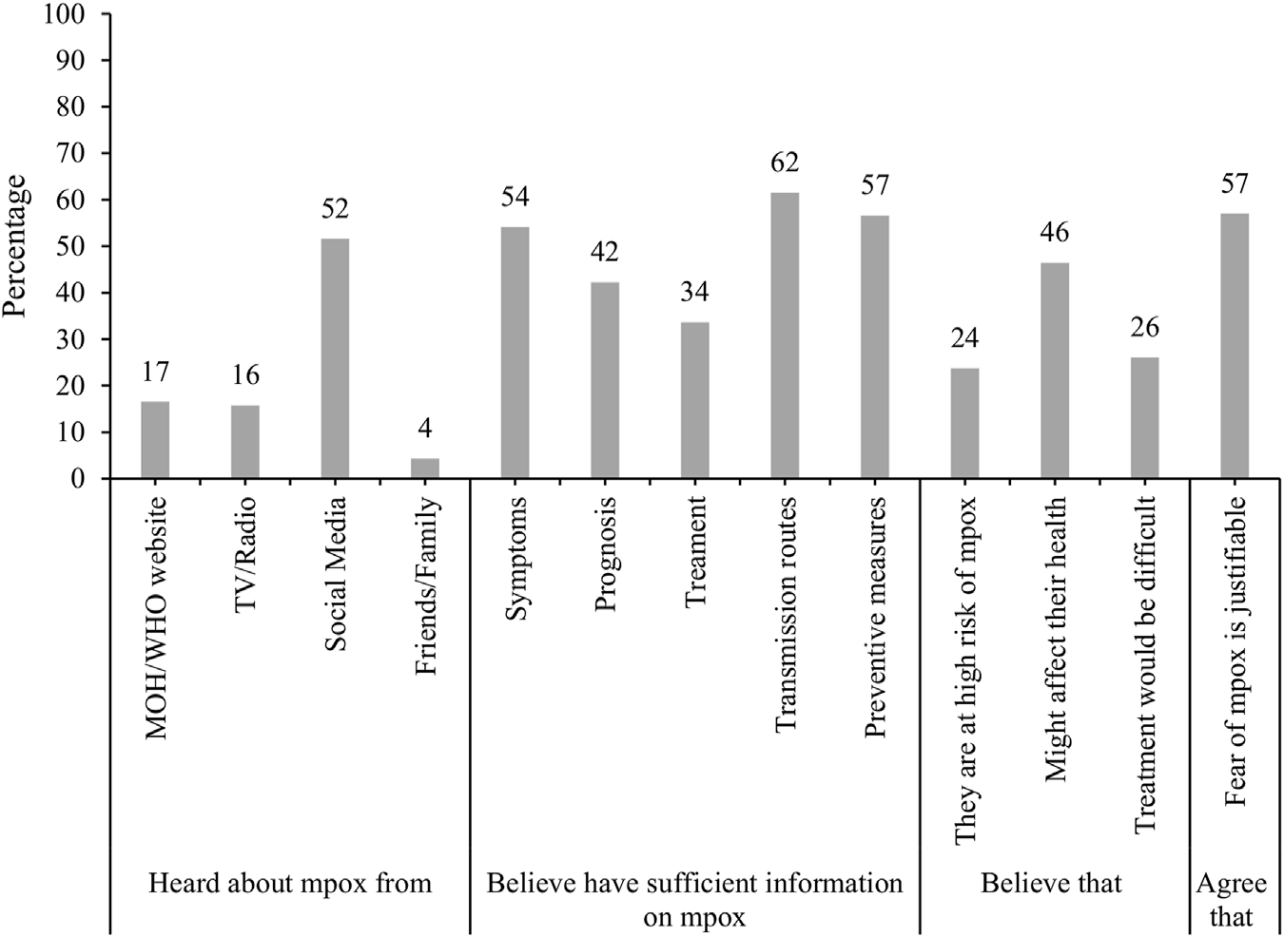
Sources of Mpox information, perceptions of knowledge and perceived risks of Mpox (N = 959). Assessment of knowledge, perceptions and attitudes during the global Mpox outbreak in June 2022: A cross-sectional study from the United Arab Emirates, United Arab Emirates, 2022.

Participants responses on each item of the knowledge questions are presented in Figure 1. A high percentage of participants knew that the transmission of mpox was possible from human to human (91.3%), and that the disease could cause rashes and fever. However, only 36.1% and 24.4% replied that it can cause death, and the possibility of catching the disease by eating wild animals, respectively.

**FIGURE 2:**
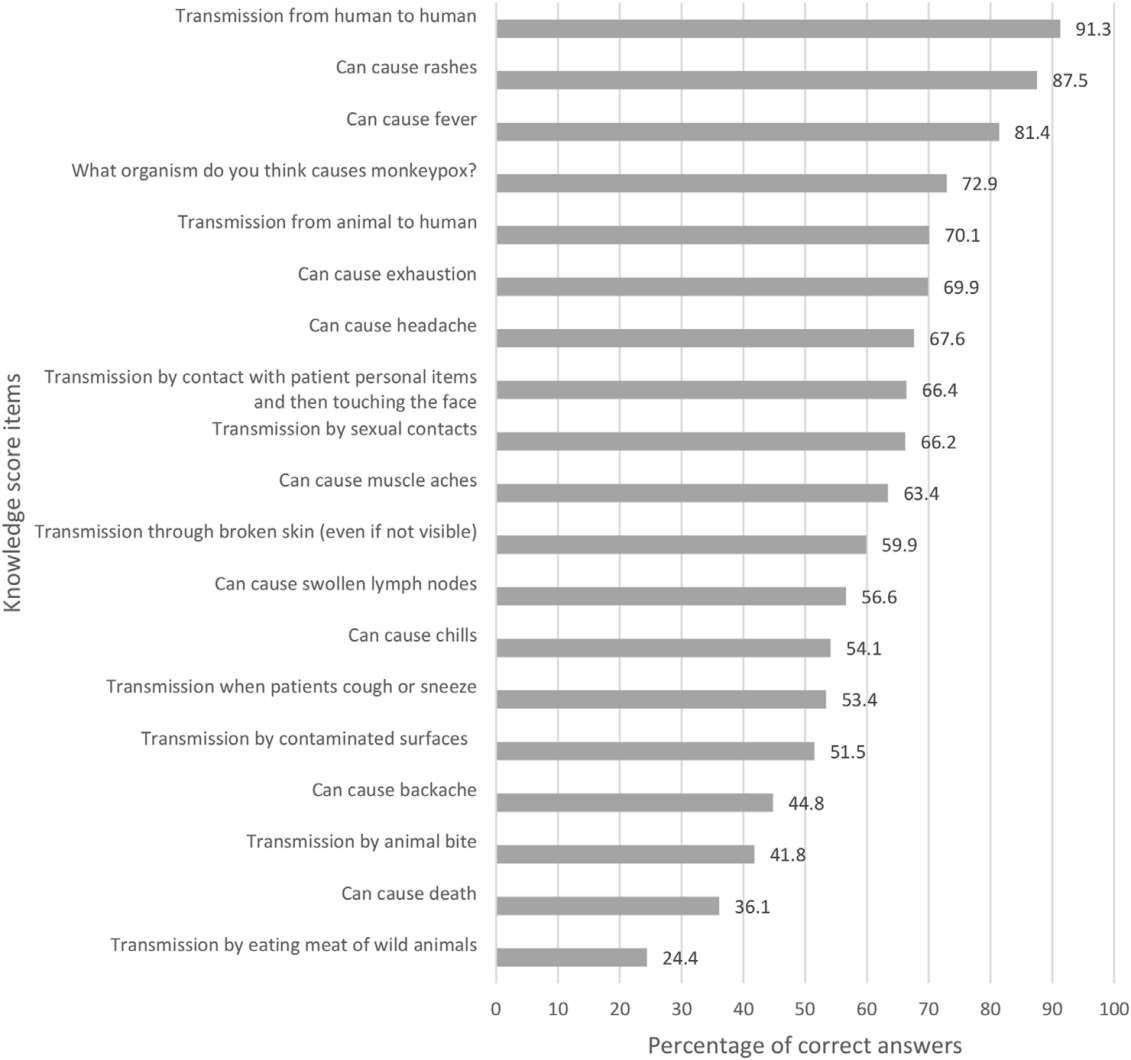
Percentage of respondents providing correct answer on each item of the knowledge score items, (N = 850). Assessment of knowledge, perceptions and attitudes during the global Mpox outbreak in June 2022: A cross-sectional study from the United Arab Emirates, United Arab Emirates, 2022.

The majority of respondents reported they had heard about mpox for the first time via social media (52%) followed by MOHAP/WHO official websites (17%) and through TV/Radio (16%). Most participants believed they had sufficient information on mpox transmission routes (62%), preventive measures (57%) and symptoms (54%). Whilst only a quarter of participants believed that they were at high risk of mpox infection, almost half (46%) believed that it might have a major effect on their health if infected. The majority of participants (57%) agreed that the fear of mpox was justified (Figure 2).

**FIGURE 3:**
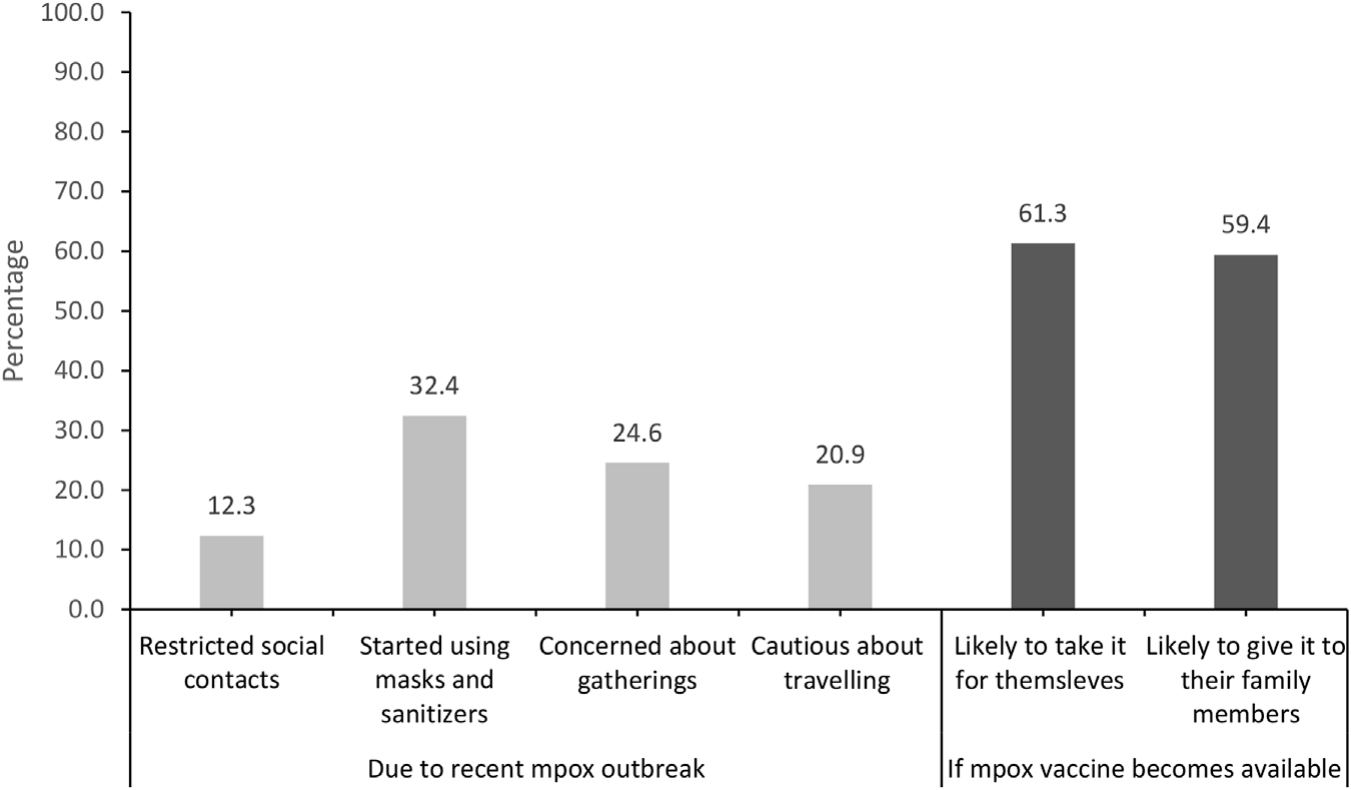
Changes in behaviour due to Mpox outbreak and likelihood to take the vaccine if available. Assessment of knowledge, perceptions and attitudes during the global Mpox outbreak in June 2022: A cross-sectional study from the United Arab Emirates, United Arab Emirates, 2022.

Figure 3 shows that 12.3% of participants reduced social contacts due to the mpox outbreak, while 32.4% resumed using masks and sanitizers. Almost a quarter of study participants (24.6%) were concerned about large gatherings and 20.9% were cautious about travel. If available, 61% admitted they would take the mpox vaccine, and 59% would give it to family.

Table 2 shows associations between respondent characteristics and mpox knowledge, worry, anxiety, and vaccine openness. Age was significantly associated with all four variables, with 35–44 year-olds having higher levels of knowledge (66%) when compared to younger participants (18–24 years) and older participants (45+ years). Worry and anxiety seemed to be lower in those who were older than 35, and they were also less likely to take the vaccine (*p* = 0.017). Knowledge levels were significantly higher in women (58%) compared to men (51%). Similarly, women were more anxious (21%) as compared to men (15%). Using the WHO or the ministry of health as a source of information, being a healthcare worker and having postgraduate degrees were associated with high knowledge scores (*p* = 0.021, *p* < 0.001, and *p* = 0.013, respectively) compared to non-HCWs and having a high school level or less. Having postgraduate degrees was also linked to less worry (*p* = 0.004) when compared to those having an undergraduate degree, and less anxiety (*p* = 0.003) when compared to the other two groups; however, they were less open to receiving the vaccine when compared to the others (*p* = 0.019).

**TABLE 2:** Knowledge, worry and anxiety levels and chi-square association by sociodemographic characteristics, (N = 959). Assessment of knowledge, perceptions and attitudes during the global Mpox outbreak in June 2022: A cross-sectional study from the United Arab Emirates, United Arab Emirates, 2022.

Variables		Knowledge score ≥12			Worry index ≥13.5			Anxiety (GAD-7 ≥ 8)				Likely to get vaccinated		
		n	%	p*	n	%	p*	n	%		p*	n	%	p*
Age				0.015			0.001				0.012			0.017
18–24		169	51%		172	53%		75	23%			207	66%	
25–34		128	59%		121	56%		43	20%			138	64%	
35–44		109	66%		80	48%		31	19%			86	53%	
45+		72	53%		47	35%		13	10%			72	55%	
Gender				0.048			0.326				0.034			0.807
Female		351	58%		302	51%		125	21%			353	61%	
Male		127	51%		118	47%		37	15%			150	62%	
Marital status				0.089			0.384				0.039			0.065
Married		239	60%		188	48%		62	16%			220	57%	
Never married		231	53%		226	52%		98	23%			275	65%	
Widowed/Divorced/Separated		8	62%		6	46%		2	15%			8	62%	
Occupation				<0.001			<0.001				<0.001			0.596
Other		329	49%		299	45%		137	21%			392	61%	
Healthcare worker		149	83%		121	68%		25	14%			111	63%	
Education				0.013			0.004				0.003			0.019
High school or less		90	48%-		87	48%		47	26%+			111	63%+	
Undergraduate or equivalent		240	56%		235	55%+		85	20%+			269	65%+	
Postgraduate Masters/PhD		148	63%+		98	42%-		30	13%-			123	54%-	
UAE National				0.356			0.576				0.006			0.850
No		364	57%		319	51%		108	17%			379	62%	
Yes		114	54%		101	48%		54	26%			124	61%	
Ethnicity				0.039			<0.001				0.303			0.236
Non-Arab		149	62%		146	61%		41	17%			151	65%	
Arab		329	54%		274	46%		121	20%			352	60%	
Source of info				0.021			0.716				0.479			0.507
Other source of info		70	48%		75	51%		31	22%			83	59%	
WHO/MOH		408	58%		345	50%		131	19%			420	62%	
Previous COVID-19 infection				0.631			0.380				0.016			0.076
No		216	57%		183	48%		76	20%			243	64%	
Yes, with no/mild symptoms		108	55%		97	49%		26	13%			108	55%	
Yes, with moderate/severe symptoms		143	59%		130	54%		58	24%			152	63%	
Believe they are at high risk of mpox				0.001			<0.001				<0.001			<0.001
Disagree/Neutral		335	54%		247	40%		99	16%			362	58%	
Agree		132	68%		163	84%		61	31%			141	72%	
Believe mpox might have major effects on their health				0.007			<0.001				<0.001			0.002
Disagree/Neutral		231	53%		151	34%		58	13%			248	56%	
Agree		236	62%		259	68%		102	27%			255	67%	
Believe treatment would be difficult				0.074			<0.001				<0.001			0.010
Disagree/Neutral		334	55%		254	42%		89	15%			356	59%	
Agree		133	62%		156	73%		71	33%			147	69%	
Agree that fear is justifiable				0.003			<0.001				<0.001			<0.001
Disagree/Neutral		178	51%		109	31%		47	13%			165	47%	
Agree		289	61%		301	64%		113	24%			338	72%	
Had a change in their behaviour following mpox outbreak				<0.001			<0.001				<0.001			<0.001
No change		243	50%		181	37%		63	13%			267	55%	
Any change		224	67%		229	69%		97	29%			236	71%	
Knowledge scores							0.002				0.865			0.344
<12.00					160	44%		69	19%			210	59%	
12.00+					260	55%		93	20%			293	63%	
Worry index											<0.001			<0.001
<13.50								36	9%			216	53%	
13.50+								126	30%			287	70%	
GAD														0.380
No GAD												400	61%	
GAD												103	64%	
COVID-19 vaccination status														<0.001
Less than two doses												12	31%	
Only two doses												100	58%	
Booster dose												391	64%	

Symbols + and – represent significant differences in *post hoc* analyses.

MOH, ministry of health; WHO, World health organization; UAE, United Arab Emirates; GAD, generalized anxiety disorder.

*Using Chi-Square test.

Anxiety levels were not significantly different between those who had moderate to severe symptoms of COVID-19 (24%) and those who had never caught the infection (20%). However, those who had contracted the virus and had no or only mild symptoms were significantly less anxious (13%) than the other two groups.

Those who believed they were at high risk of acquiring mpox, those who believed it could have major effects on their health or that the treatment would be difficult had higher knowledge scores, more worry, anxiety and openness to be vaccinated than those who did not. Similar results were also found in those who agreed that fear of mpox was justifiable.

Additionally, superior knowledge scores were associated with more worry among participants (55% with knowledge scores ≥12 vs. 44% for knowledge score <12, *p* = 0.002). Being more worried was also linked to having significantly more anxiety (*p* < 0.001) and willingness to take the vaccine (*p* < 0.001).

Table 3 displays results from the multivariable binary logistic regression analysis for the four dependent variables. The sample size for regression models was 820, as these participants had complete information on all variables included in the regression models. Women (AOR: 1.50; 95% CI: 1.08, 2.08), HCWs (AOR: 4.65; 95% CI: 3.00–7.20), and those using reliable sources of information (AOR: 1.48; 95% CI: 1.00–2.19) were more likely to have high levels of knowledge.

**TABLE 3:** Multivariate binary logistic regression analysis for the association between participants’ sociodemographic characteristics and knowledge scores (N = 820). Assessment of knowledge, perceptions and attitudes during the global Mpox outbreak in June 2022: A cross-sectional study from the United Arab Emirates, United Arab Emirates, 2022.

		AOR	95% CI[LL, UL]	*p*
Model 1: Knowledge				
Women (vs. Men)		1.50	[1.08, 2.08]	0.016
Healthcare Worker (vs. not HCW)		4.65	[3.00, 7.20]	<0.001
Education				
High school or less		1		
Undergraduate or equivalent		0.84	[0.57; 1.22]	0.359
Postgraduate Masters/PhD		1.37	[0.90; 2.10]	0.142
Source of info: WHO/MOH (vs. other)		1.48	[1.00, 2.19]	0.048
Believe mpox might have major effects on their health		1.36	[0.99, 1.86]	0.055
Had a change in their behavior		1.62	[1.18, 2.23]	0.003
Model 2: Worry				
Age				
18–24		1		
25–34		0.72	[0.46, 1.12]	0.149
35–44		0.60	[0.37, 0.96]	0.033
45+		0.41	[0.24, 0.68]	<0.001
Healthcare Worker (vs. not HCW)		2.44	[1.57, 3.80]	<0.001
Arab (vs. Not Arab)		0.67	[0.46, 0.99]	0.044
Believe they are at high risk of mpox		3.21	[2.01, 5.14]	<0.001
Believe mpox might have major effects on their health		1.83	[1.27, 2.63]	0.001
Believe treatment would be difficult		1.46	[0.95, 2.26]	0.086
Agree that fear is justifiable		2.53	[1.81, 3.52]	<0.001
Had a change in their behavior		1.93	[1.37, 2.71]	<0.001
Model 3: Anxiety				
Healthcare Worker (vs. not HCW)		0.47	[0.28; 0.79]	0.005
Education				
High school or less		1		
Undergraduate or equivalent		0.74	[0.46; 1.17]	0.195
Postgraduate Masters/PhD		0.50	[0.29; 0.88]	0.016
Previous COVID-19 infection				
No		1		
Yes, with no/mild symptoms		0.58	[0.35; 0.97]	0.037
Yes, with moderate/severe symptoms		1.00	[0.66; 1.54]	0.984
Believe treatment would be difficult		1.69	[1.13; 2.52]	0.011
Had a change in their behavior		1.94	[1.31; 2.87]	<0.001
Higher worry index		3.83	[2.47; 5.94]	<0.001
Model 4: Likeliness to take the vaccine				
Age				
18–24		1		
25–34		1.03	[0.7; 1.52]	0.890
35–44		0.59	[0.39; 0.89]	0.012
45+		0.68	[0.43; 1.05]	0.082
COVID-19 vaccination status				
Less than two doses		1		
Only two doses		2.27	[1.04; 4.94]	0.038
Booster dose		3.54	[1.71; 7.33]	<0.001
Agree that fear is justifiable		2.37	[1.73; 3.25]	<0.001
Had a change in their behavior		1.48	[1.07; 2.04]	0.019
Higher worry index		1.41	[1.02; 1.95]	0.038

HCW, healthcare worker; MOH, ministry of health; WHO, World health organization.

AOR: adjusted odds ratio; LL and UL: the lower and upper limits of a confidence interval, respectively

Model 1: Nagelkerke R2 = 0.151; Hosmer and Lemeshow Test, *p* = 0.530; Omnibus Tests of Model Coefficients, *p* < 0.001

Model 2: Nagelkerke R2 = 0.352; Hosmer and Lemeshow Test, *p* = 0.289; Omnibus Tests of Model Coefficients, *p* < 0.001

Model 3: Nagelkerke R2 = 0.206; Hosmer and Lemeshow Test, *p* = 0.853; Omnibus Tests of Model Coefficients, *p* < 0.001

Model 4: Nagelkerke R2 = 0.142; Hosmer and Lemeshow Test, *p* = 0.689; Omnibus Tests of Model Coefficients, *p* < 0.001

Worry decreased significantly after the age of 35 and was the lowest in those above 45 years (AOR: 0.41; 95% CI: 0.24–0.68). However, the odds of having high levels of worry were higher among HCWs (AOR: 2.44; 95% CI: 1.57–3.80), and among participants who believed that fear from mpox was justifiable (AOR: 2.53; (95% CI: 1.81, 3.52), believed they were at high risk of mpox infection (AOR: 3.21; 95% CI: 2.01, 5.14), and those who believed mpox infection would have major effects on their health (AOR: 1.83; 95% CI: 1.27, 2.63).

Participants who had higher levels of education were less likely to report higher anxiety levels (AOR: 0.50; 95% CI: 0.29, 0.88). Additionally, participants who had previously experienced no or mild symptoms from COVID-19 infection had lower odds of anxiety (AOR: 0.58; 95% CI: 0.35, 0.97) when compared to those who had never been diagnosed with COVID-19. However, those who believed treatment would be difficult and who were more worried reported higher levels of anxiety (AOR: 1.69; 95% CI: 1.13, 2.52 and AOR: 3.83; 95% CI: 2.47, 5.94, respectively).

As for vaccine acceptance, participants between 35–44 years of age showed less openness to take a vaccine than younger participants (AOR: 0.59; 95% CI: 0.39, 0.89). Also, respondents who had already taken two doses or a booster dose of the COVID-19 vaccine were more likely to receive the mpox vaccine (AOR: 2.27; 95% CI: 1.04, 4.94 and AOR: 3.54; 95% CI: 1.71, 7.33, respectively). Experiencing higher worry about mpox, having done changes to their behaviour as preventive measure, and having the belief that fear of mpox was justifiable were all linked to the likeliness of receiving the vaccine (AOR: 1.41; 95% CI 1.02, 1.95, AOR: 1.48; 95% CI: 1.07, 2.04, and AOR: 2.37; 95% CI, 1.73, 3.25, respectively).

## Discussion

To the best of our knowledge, this study is the first to examine knowledge, worry, and anxiety among UAE adults associated with the recent global mpox outbreak, and to explore the UAE population’s willingness to receive the mpox vaccine once available.

### Knowledge on Information Related to Mpox

The present study findings suggest that almost half of the survey participants scored less than 12 out of a maximum score of 19, showing that they could correctly answer less than 60% of the questions concerning mpox transmission, signs and symptoms. These findings are consistent with the findings from a study conducted in Saudi Arabia which reported that 52% of participants had an inadequate level of knowledge about mpox, answering 61% of questions correctly [20]. The findings have also shown that, in the UAE, HCWs were almost five times more likely to have high knowledge levels as compared to individuals from the community. Still, their mean knowledge score was of 14.4/19 (result not shown) showing room for improvement. Studies from countries like Indonesia and Italy too indicate that the knowledge of mpox transmission, symptoms and treatment is not universal among general practitioners and medical professionals [21, 22]. This may be explained by their reduced exposure to the disease due to the scarcity of mpox cases. For example, a study from Indonesia discovered that general practitioners had inadequate understanding of the new Zika virus, that has not yet been documented there. In contrast, when it came to endemic diseases in Indonesia, the level of knowledge was relatively high, even community members (66.5%) had a good understanding of dengue, a disease spread by *Aedes Aegypti* [21].

Previous empirical research has demonstrated that people with strong health knowledge exhibit a better understanding of health topics, increasing their likelihood of expressing better health behaviours [23, 24]. In a systematic review, adopting healthy habits and practices was significantly associated with improved health knowledge during pandemic emergencies such as H1N1 and COVID-19 [25]. Almost half of our sample believed that mpox might have major effects on their health, and this belief was associated with higher knowledge levels. Having adequate knowledge also helps in curbing the spread of misinformation and rumours related to the disease. The majority of participants in our study learned about mpox from social media and very few accessed information from official sources such as the Ministry of Health/WHO or TV/radio. The experience gained during the COVID-19 pandemic indicates that exposure to traditional media (TV/radio/newspapers) is associated with lower misinformation beliefs, while exposure to digital media and personal contacts is associated with greater conspiracy and misinformation beliefs [26]. Misinformation beliefs can induce panic and stress among people, similar to the effect of the spread of misinformation related to COVID-19 [12].

### Change in Behavior

Although around 60% of the respondents agreed that the fear of mpox was justified, relatively low numbers reported they had made any behavioural changes to prevent infection such as restricting their social contacts, wearing masks, using sanitizers, and refraining from large gatherings due to the mpox outbreak. We could not ask the participants about changes in their sexual contact behavior due to cultural sensitivities in the UAE. Twenty to thirty percent of the participants in this study reported taking protective measures like masks and sanitizers more seriously, with 24% expressing a belief in being at high risk of contracting the virus. These findings reflect concerns of the perceived risk despite the underreported cases of mpox during the study period. Authorities can build upon these findings, focusing on the education of health workers to effectively disseminate accurate information about mpox prevention strategies. It is important to note that the modes of transmission for mpox do not align with the conventional use of masks and sanitizers. Hence, health workers should emphasize strategies like early detection, reporting, and appropriate quarantine measures to curb the spread of mpox.

### Worry and Anxiety

The emergence of mpox cases in several countries has focused attention on the topic of psychosocial consequences [6]. Studies in the UAE and region conducted during the COVID-19 pandemic showed an increase in mental health disorders due to fear of the new disease and social restrictions imposed during the pandemic [14, 27, 28]. Our study found people to be worried and anxious about mpox. Public’s anxiety and worry are understandable for many reasons, including the fact that mpox emerged during the COVID-19 pandemic, and that it is new to the UAE, which was the first in the Middle East to report it [29].

With regards to factors associated with anxiety, highly educated individuals reported lower anxiety levels in our study. The role of education in protecting against anxiety were also documented in a previous study [30]. It is believed that people with greater levels of education will have access to more reliable psychosocial resources that will enable them to manage with anxieties brought on by mpox.

Another interesting, identified factor for anxiety was that respondents who had no or only mild symptoms when infected with SARS-CoV-2 were less anxious about mpox than those who had not been diagnosed with the virus. This suggests that experience of mild symptoms of COVID-19 can alleviate fears about future outbreaks, while severe symptoms have been linked to PTSD and higher anxiety levels in survivors [31, 32]. On the other hand, worry was identified in the present study to be a risk factor for anxiety. Many previous studies have reported that history of previous psychiatric illness is associated with high level of anxiety and mental health disorders [33, 34]. In fact, worrying is considered an essential component of many kinds of anxiety disorders [35, 36]. Previous work has also emphasized the impact of worry about a certain event can be equal to or even more than experiencing an event [37]. These results may highlight the possible bidirectional process between experiencing worries and becoming more anxious [36]. Concerning factors associated with worry, our results show that worry about mpox declined with age and was lowest in individuals over the age of 45 years; despite the fact that the older persons are more susceptible to developing complications [38, 39] Previous studies have reported similar results [40, 41]. One study on COVID-19 worries and mental health, found that older age moderated the impact of COVID-19 by acting as a buffer against the negative psychological effects [32]. A possible explanation for this finding is that older adults are more capable of adapting to stressors than younger adults; therefore, their perceived stress decreases with age [42, 43].

In our study, being a healthcare worker (HCW) was found to be associated with greater concerns about mpox compared to non-HCWs. This finding is consistent with previous pandemic outbreaks, such as COVID-19 [44, 45] and SARS [46]. The assumption is that due to their close contact with patients and extended working hours, HCWs perceive themselves to be at higher risk of contracting mpox. However, in the case of mpox WHO has reported that most infections among HCWs occurred in the community, rather than from occupational exposure [3]. Nevertheless, the emotional exposure that HCWs undergo while witnessing patients’ suffering, worry, pain, hardship, and even death might contribute to their heightened concerns about mpox, as opposed to non-HCWs.

Individuals who believed that they were at a high risk of contracting mpox, and who were concerned about its detrimental effects on their health scored higher on worry measures. These findings provide a backdrop of possible reactions to a potential outbreak in the UAE and will be (are) helpful in preparing for future outbreaks.

Another factor was that individuals who believed that fear of mpox was justified had higher levels of worry. Exploring the reasons behind this fear was not within the scope of this study. However, it would be interesting to investigate in future studies, as it may be linked to an individual’s own risk of contracting mpox. Stigma related to mpox could also play a role in anxiety and fear. Al Hammadi et al. were interested in the clinical characteristics of mpox infection in patients from two centers in Abu Dhabi, UAE [4]. Forty percent of their patients reported sexual contact within 21 days of symptom onset, and none reported same-sex sexual contact. The study found sexual exposure to be a significant factor in mpox infection cases but noted a reluctance among patients to disclose such information, likely due to cultural and religious factors as well as the stigma associated with mpox infection. This lack of disclosure and the associated stigma may hinder accurate diagnosis and disease control measures; it also contributes to the scarcity of knowledge concerning the disease in the Middle East. In order to address this stigma and discrimination, the WHO created a guidance document providing insights on recommended language and actions to be used to counter stigmatization, aimed at the different actors and stakeholders [47]. Other resources were also created for people who are more at risk of stigmatization [48, 49].

### Vaccine Acceptance

Three vaccines have been approved for use in mpox prevention [50]. However, the interim guidance, developed by the WHO with the advice and support of the Strategic Advisory Group of Experts (SAGE) Working Group on smallpox and monkeypox vaccines, recommended that mass vaccination was neither required nor recommended. Instead, primary preventive vaccination was recommended for individuals at risk [50]. The WHO also left it to countries to determine their clinical and public health needs regarding vaccines. Even without the requirement for mass vaccination, it would be interesting to explore the participants’ openness to the idea of receiving the mpox vaccine, as this would provide an indication of their potential reaction in future outbreaks. Although there were no significant changes in preventive measures in their daily life, a substantial proportion (61%) of the participants expressed willingness to receive the mpox vaccine if available. This is a higher percentage than in Saudi Arabia, where only 50.6% favored vaccination [51]. It is noteworthy that both studies have comparable populations, as they are both online surveys conducted in GCC countries with a high large expatriate population. Previous vaccination behavior seems to positively influence mpox vaccine acceptance, as those who received COVID-19 booster shots were more likely to get vaccinated and encourage family members to do so. Other studies on COVID-19 and mpox vaccine acceptance have found similar predictors of vaccine acceptance, including perceived risk, vaccine efficacy, personal experiences with COVID-19, and its vaccination [51–53]. Older adults were less willing to receive the mpox vaccine, consistent with findings from [51].

### Limitations

Findings should be interpreted in light of some limitations. First, the study’s cross-sectional design, which only permits correlational, not causational, inference due to temporality. Given the utilization of convenience sampling and the online survey format, selection bias is likely. The demographic composition of our sample differs from the national distribution. Our study sample exhibits a higher ratio of women to men, is generally younger compared to the wider population, and includes a larger proportion of healthcare workers. This can be attributed to the nature of online surveys, which tend to be more accessible to younger individuals with internet access and technological proficiency. The higher representation of healthcare workers in our sample is a result of utilizing the author’s professional network as the starting point for snowball sampling. This may hinder the generalizability of the scores. Hence, it is important to note that these survey findings offer an understanding into the experiences, beliefs, and attitudes towards mpox in the country in the early days, and extrapolation to the whole population should be done with caution. It is worth highlighting that the terms “belief,” “fear,” “worry,” and “anxiety” were used in a general sense throughout the study, without providing specific operational definitions to the participants. Hence, individuals may have interpreted these questions very differently. Additionally, the use of GAD-7 does not allow for clinical diagnosis, but rather a screening to flag anxiety levels for further investigation. Moreover, this study does not allow for assessing worry and anxiety in relation to the actual risk of contracting the disease, as no questions were asked regarding the respondents’ protective or risky behaviors. However, this study aimed to explore the perceived risk, based on the subjective judgment of individuals. In addition, some questions could have been misunderstood and introduced an information bias. Generalizability of the findings should be done with caution while considering these limitations.

### Conclusion

This study highlights the connection between risk perception, knowledge, worry, anxiety, and vaccine acceptance regarding mpox. The level of knowledge about mpox was not consistent among the surveyed population. When mpox cases were identified in the UAE, the Ministry of Health and Prevention (MoHAP) swiftly implemented precise mechanisms for identifying and confirming suspected mpox cases and closely monitoring contacts of patients. They also disseminated a comprehensive guide for surveillance, early detection, patient management, and precautionary measures to stakeholders and through various media platforms [54]. However, the lack of knowledge, even among healthcare workers, suggests room for improvement. To address this, targeted awareness campaigns on social media platforms should be launched during similar public health emergencies. Collaborating with trusted online communities and influencers to share reliable information can be a key strategy. Considering it is the age of the digital information by implementing these recommendations, social media can be leveraged as a powerful tool to increase knowledge about mpox and future disasters, while combating misinformation and reducing unnecessary worry. A dedicated team should actively monitor, and address misinformation related to such outbreaks on social media.

This study also revealed that nearly half of the sample expressed concern about the potential impact of mpox on their health and lives, leading to increased worry and anxiety. Like previous pandemics, low knowledge and misinformation can contribute to worry and anxiety. Therefore, the public health sector should prioritize the dissemination of accurate information through multiple platforms and in multiple languages. Educational materials such as brochures, pamphlets, posters, and infographics can be utilized to explain mpox, its transmission, preventive measures, and steps to take in case of suspected infection. Furthermore, the study also found that healthcare workers had a higher level of worry compared to the general population. It is important to address their concerns and prioritize their wellbeing by implementing comprehensive mental health support programs and providing training sessions to update healthcare workers on the latest information about the pandemic.
